# Expression of the α7 nicotinic acetylcholine receptor in human lung cells

**DOI:** 10.1186/1465-9921-6-29

**Published:** 2005-04-04

**Authors:** Howard K Plummer, Madhu Dhar, Hildegard M Schuller

**Affiliations:** 1 Molecular Cancer Analysis Laboratory, Department of Pathobiology, College of Veterinary Medicine, University of Tennessee, Knoxville, TN 37996-4542, USA; 2 Experimental Oncology Laboratory, Department of Pathobiology, College of Veterinary Medicine, University of Tennessee, Knoxville, TN 37996-4542, USA

## Abstract

**Background:**

We and others have shown that one of the mechanisms of growth regulation of small cell lung cancer cell lines and cultured pulmonary neuroendocrine cells is by the binding of agonists to the α7 neuronal nicotinic acetylcholine receptor. In addition, we have shown that the nicotine-derived carcinogenic nitrosamine, 4(methylnitrosamino)-1-(3-pyridyl)-1-butanone (NNK), is a high affinity agonist for the α7 nicotinic acetylcholine receptor. In the present study, our goal was to determine the extent of α7 mRNA and protein expression in the human lung.

**Methods:**

Experiments were done using reverse transcription polymerase chain reaction (RT-PCR), a nuclease protection assay and western blotting using membrane proteins.

**Results:**

We detected mRNA for the neuronal nicotinic acetylcholine receptor α7 receptor in seven small cell lung cancer (SCLC) cell lines, in two pulmonary adenocarcinoma cell lines, in cultured normal human small airway epithelial cells (SAEC), one carcinoid cell line, three squamous cell lines and tissue samples from nine patients with various types of lung cancer. A nuclease protection assay showed prominent levels of α7 in the NCI-H82 SCLC cell line while α7 was not detected in SAEC, suggesting that α7 mRNA levels may be higher in SCLC compared to normal cells. Using a specific antibody to the α7 nicotinic receptor, protein expression of α7 was determined. All SCLC cell lines except NCI-H187 expressed protein for the α7 receptor. In the non-SCLC cells and normal cells that express the α7 nAChR mRNA, only in SAEC, A549 and NCI-H226 was expression of the α7 nicotinic receptor protein shown. When NCI-H69 SCLC cell line was exposed to 100 pm NNK, protein expression of the α7 receptor was increased at 60 and 150 min.

**Conclusion:**

Expression of mRNA for the neuronal nicotinic acetylcholine receptor α7 seems to be ubiquitously expressed in all human lung cancer cell lines tested (except for NCI-H441) as well as normal lung cells. The α7 nicotinic receptor protein is expressed in fewer cell lines, and the tobacco carcinogen NNK increases α7 nicotinic receptor protein levels.

## Background

We and others have shown that one of the mechanisms of growth regulation of small cell lung cancer (SCLC) cell lines and cultured pulmonary neuroendocrine cells (PNEC) is by the binding of agonists to a cell surface receptor of the neuronal nicotinic acetylcholine receptor family comprised of homomeric α7 subunits, which functions as an ion channel with high permeability for Ca^2+ ^[1-8]. Binding of agonists to this receptor activates the release of the autocrine growth factor serotonin [2-6]. In addition, we have shown that the nicotine-derived carcinogenic nitrosamine, 4(methylnitrosamino)-1-(3-pyridyl)-1-butanone (NNK), is a high affinity agonist for the α7 nicotinic acetylcholine receptor (α7 nAChR) [5]. Binding of NNK to this receptor caused an influx of Ca^2+ ^from the extracellular environment [7], resulting in the activation of a protein kinase C-dependent Raf-1/MAP kinase-mediated mitogenic pathway [5,9,10]. These findings suggest that the chronic stimulation of this pathway may contribute to the selective development of this histologic cancer type in smokers. Accordingly, components of this signal transduction pathway may be promising targets for cancer intervention studies with selectivity for SCLC.

Our goal in the present studies was to determine the extent of α7 mRNA expression in the human lung to determine if previous research with SCLC could be extrapolated to other lung malignancies. Previous research in our laboratory indicated expression of mRNA for the α7 receptor in normal fetal hamster PNEC cells [6]. PNEC cells are one of the possible cells of origin for SCLC [11,12]. We screened multiple small cell and non-small cell lung cancer cell lines, normal cells, and fresh surgical tissue samples from cancer patients for mRNA expression of the α7 receptor. With the exception of NCI-H441 cell line, all cell lines and patient samples tested expressed mRNA for the α7 receptor, and the α7 nicotinic receptor protein is expressed in fewer cell lines.

## Methods

### Cell culture

The human SCLC cell lines NCI-H69, NCI-H82, NCI-H146, NCI-H187, NCI-H209, and NCI-H526, the human adenocarcinoma cell lines NCI-H322 and NCI-H441, and A549, the carcinoid cell line NCI-H727, and the squamous cell lines NCI-H226, NCI-H2170, and NCI-H520 were purchased from the American Type Culture Collection (Manassas, VA). The human SCLC cell line WBA [13] was a gift of Dr. G. Krystal, Medical College of Virginia. All cancer cell lines except A549 were maintained in RPMI medium supplemented with fetal bovine serum (10% v/v), L-glutamine (2 mM), penicillin (100 U/ml) and streptomycin (100 μg/ml) at 37°C in an atmosphere of 5% CO_2_. A549 cells were grown in Hams F12 media with supplements as above. Human small airway epithelia cells (SAEC) were purchased from Clonetics/BioWhittaker (Walkersville, MD). These cells were maintained in SAEC basal medium with supplements (Clonetics) at 37°C in an atmosphere of 5% CO_2_. Fresh surgical tissue samples were collected from patients at the University of Tennessee Graduate School of Medicine's Cancer Center and processed for reverse transcription polymerase chain reaction (RT-PCR). The collection of tissue was approved by the University of Tennessee Institutional Review Board, and the authors have been certified by the NIH Office of Human Subjects Research.

### RT-PCR

RT-PCR assays were conducted with all cells and tissues. Expression of the α7 receptor by RT-PCR in fetal hamster PNECs has been previously published [6]. RT-PCR was done as described before [6] except new human α7 primers (forward 5'-gccaatgactcgcaaccactc-3' and reverse 5'-ccagcgtacatcgatgtagca-3' bases 236–571, Genbank accession number X70297) were used. These amplified a 335 bp fragment. Oligonucleotide primers were acquired from Life Technologies (Grand Island, NY). These primer pairs are in areas of the sequence that are homologous between humans, rats, and chick brain [14]. Reactions were run on a MJ Research PTC-200 thermal cycler (Watertown, MA) with the following conditions: 1 cycle of 2 min. at 94°C, 40 cycles of 94°C, 30 sec; 55°C, 30 sec; 72°C, 45 sec, with a final extension for 5 min. at 72°C.

### Sequencing

Several PCR products were sequenced to verify the integrity of the PCR process. The NCI-H69, NCI-H322, and SAE cells were sequenced using the forward PCR primer for human α7. Sequencing was done with the ABI Terminator Cycle Sequencing reaction kit on an ABI 373 DNA sequencer (Perkin-Elmer, Foster City, CA).

### Nuclease-protection assay

The nuclease protection assay was used to determine differences in expression levels in a representative SCLC cell line (NCI-H82), and in SAE cells. The Lig'nScribe kit (Ambion, Austin, TX) was used to add a T7 RNA polymerase promoter to the PCR fragment amplified by the gene specific α7 primer, and then this T7-α7 fragment was PCR amplified, and the resulting fragment was used directly in a transcription reaction using the MAXIscript in vitro transcription kit (Ambion). This transcription reaction consisted of the PCR fragment, 10X transcription buffer, 10 mM ATP, UTP and GTP, T7 polymerase, [α-^32^P] CTP (800 Ci/mmol, Dupont-NEN, Boston, MA), and nuclease free water to a final volume of 20 μl. A probe for 28S ribosomal RNA was also transcribed and used as an internal control. These reactions were incubated for 1 hour at 37°C. After this incubation, 1 μl of DNase I was added, and the mixture was further incubated for 15 min. at 37°C. The probes were gel purified.

The RPA III kit (Ambion) was used. A molar excess of labeled probes (28S and gene specific) were added to 20 μg total RNA or Yeast RNA (Ambion), and the RNA samples and probes were co-precipitated and resuspended in hybridization buffer. After incubation at 95°C for 4 min., and incubation overnight at 42°C, RNase digestion buffer containing RNase A/RNase T1 was added. After digestion for 30 min. at 37°C, RNase inactivation/precipitation solution was added. After precipitation the pellets were resuspended in gel loading buffer, heated at 95°C for 4 min. and loaded onto a 5% polyacrylamide, 8 M urea gel (Bio-Rad, Hercules, CA). In addition RNA Century Markers Plus templates (Ambion) were transcribed and used as markers. After electrophoresis, the gel was transferred to blotting paper, dried, and exposed to Kodak XAR film.

### Western blots

Cell pellets were collected and membrane protein was isolated with the ReadyPrep protein extraction kit (signal) (Biorad). Protein levels were determined using the RCDC kit (Biorad). Aliquots of 20–30 μg protein were boiled in 3x loading buffer (New England Biolabs, Beverly, MA) for 2 minutes, then loaded onto 12% Tris-glycine-polyacrylamide gels (Cambrex, Rockland, ME), and transferred electrophoretically to nictrocellulose membranes. Membranes were incubated with the primary antibody (alpha 7 nicotinic acetylcholine receptor; Abcam, Cambridge, MA). In all western blots, membranes were additionally probed with an antibody for actin (Sigma) to ensure equal loading of protein between samples. The membranes were then incubated with appropriate secondary antibodies (Rockland, Gilbertsville, PA or Molecular Probes, Eugene OR). The antibody-protein complexes were detected by the LiCor Odyssey infrared imaging system (Lincoln, NE). Cells for some blots were incubated with 100 pM NNK (Midwest Research Institute, Kansas City, MO) for various times.

## Results

**Figure 1:**
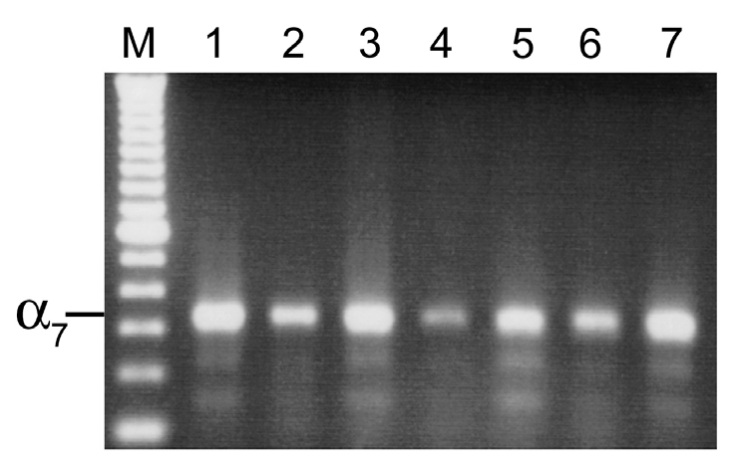
**Agarose gel showing α7 nicotinic acetylcholine receptor expression in SCLC cell lines. **cDNA was amplified by PCR using the human α7 primers. **SCLC cell lines: 1**) WBA; **2**) NCI-H69; **3**) NCI-H82; **4**) NCI-H146; **5**) NCI-H187; **6**) NCI-H209; **7**) NCI-H526. For all gene expression experiments, negative control reactions were performed and found to be negative. The bands were consistent with the expected size, 335 bp. M-100 bp DNA ladder.

**Figure 2:**
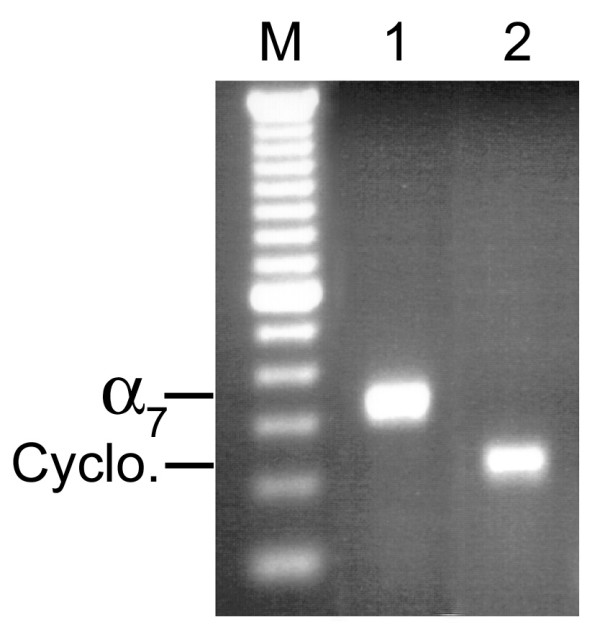
**Agarose gel showing α7 nicotinic acetylcholine receptor expression in normal small airway epithelial cells. **cDNA was amplified by PCR using the human α7 and cyclophilin primers. **1**) α7 primers; **2**) cyclophilin primers. For all gene expression experiments, negative control reactions were performed and found to be negative. The bands were consistent with the expected sizes, 335 bp for the α7 primers and 216 bp for the cyclophilin primers. M-100 bp DNA ladder.

**Figure 3:**
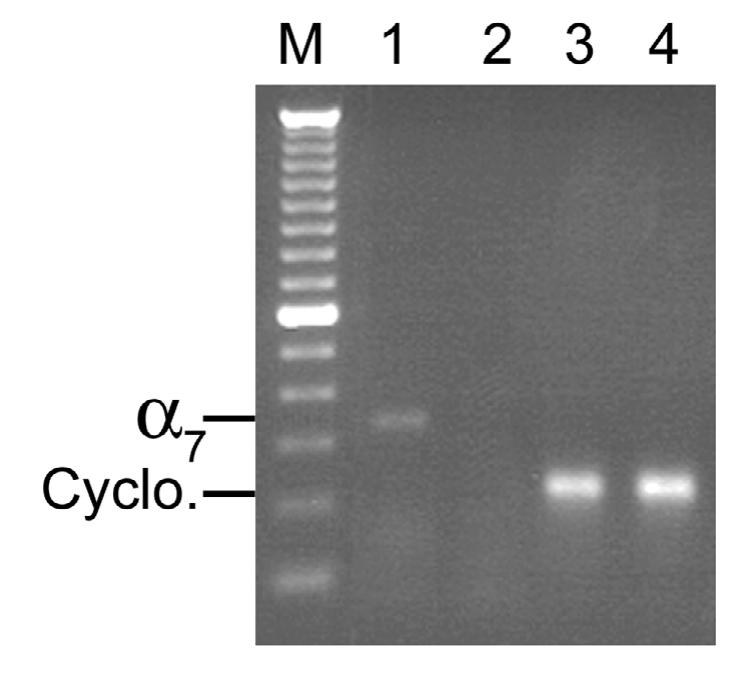
**Agarose gel showing α7 nicotinic acetylcholine receptor expression in NCI-H322 but not NCI-H441 adenocarcinoma cell lines. **cDNA was amplified by PCR using the human α7 and cyclophilin primers. **1) **NCI-H322, α7 primers; **2) **NCI-H441, α7 primers; **3) **NCI-H322, cyclophilin primers; **4) **NCI-H441, cyclophilin primers. For all gene expression experiments, negative control reactions were performed and found to be negative. The bands were consistent with the expected sizes, 335 bp for the α7 primers and 216 bp for the cyclophilin primers. M-100 bp DNA ladder.

The RT-PCR assay demonstrated expression of the α7 nAChR in all seven cultured human SCLC cell lines (Figure 1) and in SAE cells (Figure 2). Among the two adenocarcinoma cell lines, NCI-H322 demonstrated expression of the α7 nAChR, whereas NCI-H441, yielded negative results (Figure 3). Both adenocarcinoma cell lines demonstrated expression of the cyclophilin control indicating that the α7 nicotinic acetylcholine receptor was either not expressed in NCI-H441 cells, or expression levels were below the limit of detection of RT-PCR samples run on an agarose gel.

To verify the RT-PCR results, RT-PCR products from one SCLC cell line (NCI-H69) and the adenocarcinoma cell line NCI-H322 were sequenced using the forward primer used for RT-PCR amplification of the samples (data not shown). The sequences from the PCR products of both NCI-H69 and NCI-H322 were compared to the sequence of the α7 nicotinic acetylcholine receptor (bases 257–571, Genbank accession number X70297) and were found to be 100% homologous.

**Figure 4:**
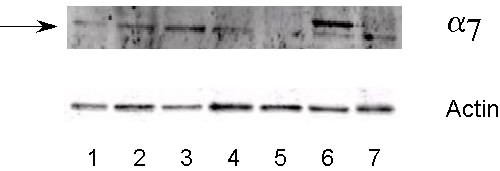
**Expression of α7 nicotinic acetylcholine receptor protein in SCLC cell lines as assessed by western blot analysis. **Protein was isolated with the ReadyPrep protein extraction kit (signal) (Biorad). Nitrocellulose membranes were incubated with the rabbit polyclonal antibody to the alpha 7 nicotinic acetylcholine receptor (Abcam). **1**) WBA; **2**) NCI-H69; **3**) NCI-H82; **4**) NCI-H146; **5**) NCI-H187; **6**) NCI-H209; **7**) NCI-H526. The arrow indicates the 56 kDa band (expected size). All SCLC cell lines except NCI-H187 express protein for alpha 7 nicotinic acetylcholine receptor.

Although we have shown mRNA expression for the α7 nicotinic acetylcholine receptor, it was necessary to show if expression of the α7 nicotinic acetylcholine receptor protein in seen in these cell lines. Using a specific antibody for the α7 nicotinic acetylcholine receptor (Abcam), membrane protein from the cell lines were assessed by western blotting. Expression of α7 protein was seen in 6 of the 7 SCLC cell lines (Figure 4). Expression of α7 protein was not seen in the NCI-H187 cell line (Figure 4). Actin levels were unchanged in all 7 SCLC cell lines (Figure 4).

**Figure 5:**
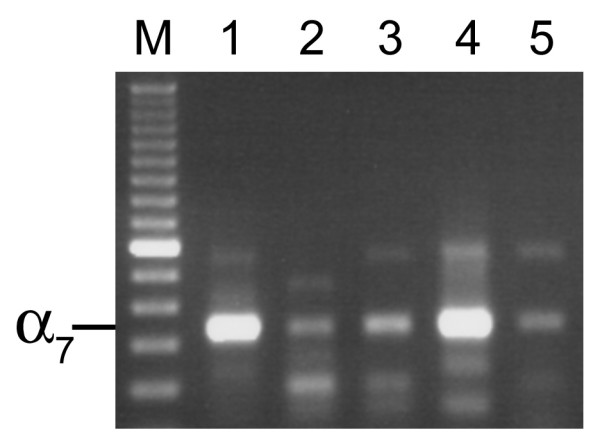
**Agarose gel showing α7 nicotinic acetylcholine receptor expression in A-549 adenocarcinoma, NCI-H727 carcinoid, and squamous cell lines NCI-H226, NCI-H520, and NCI-H2170. **cDNA was amplified by PCR using the human α7 primers. **1) **A549; **2) **NCI-727; **3) **NCI-H226; **4) **NCI-H520; **5) **NCI-H2170. For all gene expression experiments, negative control reactions were performed and found to be negative. The bands were consistent with the expected size, 335 bp. M-100 bp DNA ladder.

**Figure 6:**
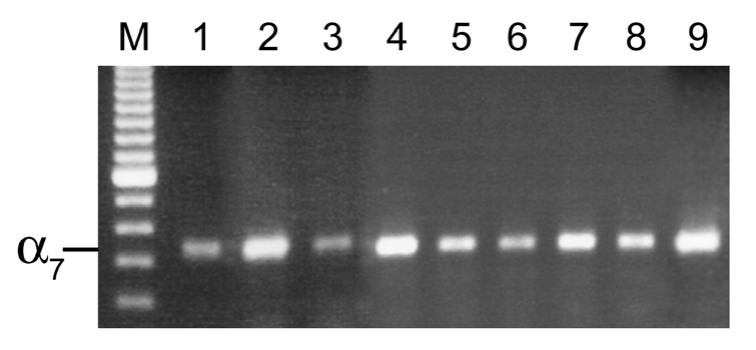
**Agarose gel showing α7 nicotinic acetylcholine receptor expression in tissue samples from lung cancer patients. **cDNA was amplified by PCR using the human α7 primers. **1) **large cell carcinoma; **2) **carcinoid; **3–5) **squamous cell carcinomas; **6–8) **adenocarcinomas; **9) **adenocarcinoma/carcinoid. For all gene expression experiments, negative control reactions were performed and found to be negative. The bands were consistent with the expected size, 335 bp. M-100 bp DNA ladder.

Additional non-small cell lung cancer cell lines were screened for the presence of α7 nAChR mRNA expression. Expression of α7 nAChR was also seen in A549 adenocarcinoma and NCI-H727 carcinoid cell lines (Figure 5). The mRNA for α7 nAChR was also expressed in three squamous cell lines, NCI-H2170, NCI-H226, and NCI-H520 (Figure 5). Expression of the α7 nAChR was also seen in nine fresh tissue samples from lung cancer patients, all of which were smokers (Figure 6).

**Figure 7:**
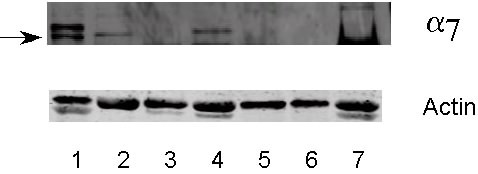
**Expression of α7 nicotinic acetylcholine receptor protein in non-SCLC cell lines and normal SAE cells as assessed by western blot analysis. **Protein was isolated with the ReadyPrep protein extraction kit (signal) (Biorad). Nitrocellulose membranes were incubated with the rabbit polyclonal antibody to the alpha 7 nicotinic acetylcholine receptor (Abcam). **1**) SAEC; **2**) A549; **3**) NCI-727; **4**) NCI-H226; **5**) NCI-H520; **6**) NCI-H2170; **7**) NCI-H322. The arrow indicates the 56 kDa band (expected size). Only SAEC, A549 and NCI-H226 express protein for alpha 7 nicotinic acetylcholine receptor.

The non-SCLC cell lines and normal cells were also screened for expression of α7 nicotinic acetylcholine receptor protein. SAEC, the adenocarcinoma cell line A549 and the squamous cell line NCI-H226 express protein for α7 (Figure 7), whereas the adenocarcinoma cell line NCI-H322, the carcinoid cell line NCI-H727, the squamous cell lines NCI-H520 and NCI-H2170 did not express protein for the α7 nicotinic acetylcholine receptor (Figure 7). Actin levels were unchanged in all protein samples (Figure 7).

**Figure 8:**
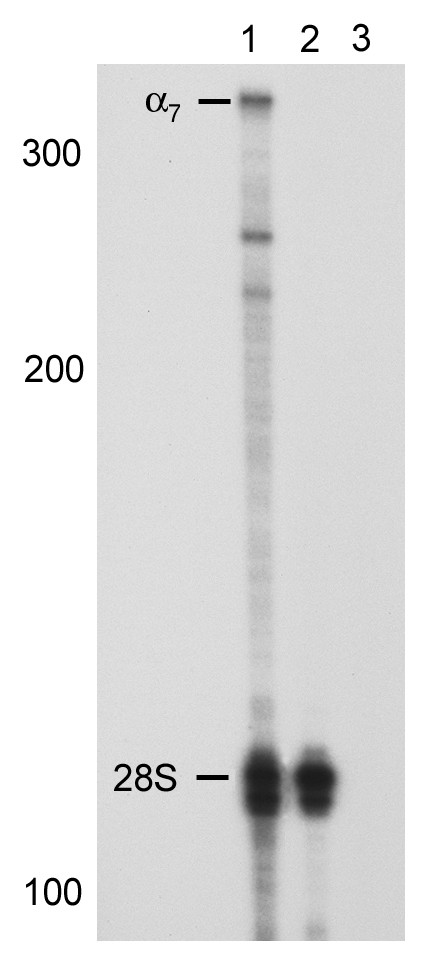
**Comparison of expression levels of α7 nicotinic acetylcholine receptor between a representative SCLC cell line (NCI-H82), and SAE cells by a nuclease protection assay. **A riboprobe transcribed from the human α7 PCR primer was used to compare expression levels in the two cell systems. **1) **NCI-H82; **2) **SAEC; **3) **Yeast RNA. The protected fragments were consistent with the expected sizes, 335 bp for the α7 primers and 115 bp for the 28S ribosomal RNA control primers. The molecular weight markers are indicated by the numbers on the left side of the figure.

To allow for a direct comparison of expression levels of α7 mRNA between a representative SCLC cell line (NCI-H82), and human SAE cells from a non-smoker (information provided by Clonetics), a riboprobe was transcribed from the PCR fragment amplified by the gene specific α7 primer used for RT-PCR and was used in a nuclease protection assay. Expression of mRNA for the α7 nicotinic acetylcholine receptor was demonstrated in the SCLC cell line NCI-H82, however no protected band was detected in the normal small airway epithelial cells (Figure 8). Expression of the 28S ribosomal RNA used as an internal control was seen in both samples (Figure 8). No protected bands were seen with either probe when annealed to yeast RNA (Figure 8). The increased levels of α7 nAChR mRNA as compared with the normal SAE cells suggest that some SCLC cells may have higher levels of α7 nAChR than normal lung epithelial cells.

**Figure 9:**
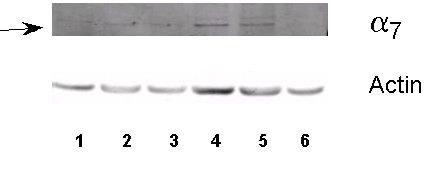
**Increases in expression of α7 nicotinic acetylcholine receptor protein in NCI-H69 after treatment with 100 pM of the tobacco carcinogen NNK as assessed by western blot analysis. **Protein was isolated with the ReadyPrep protein extraction kit (signal) (Biorad). Nitrocellulose membranes were incubated with the rabbit polyclonal antibody to the alpha 7 nicotinic acetylcholine receptor (Abcam). **1**) control; **2**) 5 min; **3**) 30 min; **4**) 60 min; **5**) 150 min; **6**) 24 hour. The arrow indicates the 56 kDa band (expected size). Increases were seen in expression of protein for alpha 7 nicotinic acetylcholine receptor after 60 min and 150 min of NNK treatment.

To determine if the α7 nicotinic acetylcholine receptor protein is a functional protein, we stimulated a representative SCLC cell line, NCI-H69 with NNK. This cell line had been used previously in our laboratories to show α7 nicotinic acetylcholine receptor specific binding [5]. The tobacco carcinogen NNK (100 pM) increased expression of α7 protein levels after 60 and 150 minutes of treatment (Figure 9). Actin levels were unchanged between the times protein was collected (Figure 9).

## Discussion

Our data supports the ubiquitous expression of the α7 nAChR mRNA in both normal and cancerous lung cells. With the exception of the NCI-H441 adenocarcinoma cell line, the α7 nAChR mRNA was expressed in all normal and cancer cells tested. Previous research in our laboratory indicated expression of mRNA for the α7 receptor in normal fetal hamster PNEC cells [6]. PNEC cells are one of the possible cells of origin for SCLC [11,12]. The expression of the α7 nAChR is not an artifact of cell culture since the expression of the α7 nAChR was seen in nine tumor samples from different patients with lung cancer. We also found that the α7 nAChR is expressed in five distinct types of cancer: squamous, carcinoid, adenocarcinoma, large cell carcinoma, and small cell lung cancer. This is the first report of the expression of the α7 nAChR receptor mRNA in pulmonary squamous, carcinoids or large cell carcinomas. A recent report indicated α7 nAChR receptor mRNA in both human bronchial epithelial cells and airway fibroblasts [15], supporting the hypothesis of ubiquitous expression of the α7 nAChR receptor mRNA in human lung cells.

In addition, we have demonstrated expression of the α7 nAChR protein of the correct molecular weight in lung cancer cells for the first time. Of the seven SCLC cell lines tested for the α7 nAChR mRNA expression, only the NCI-H187 cell line did not express a band of the correct molecular weight for the α7 nAChR. In the non-SCLC cells and normal cells that express the α7 nAChR mRNA, α7 nAChR protein expression was more limited than in SCLC. SAEC (normal cells), the adenocarcinoma cell line A549 and the squamous cell line NCI-H226 express protein for α7, whereas the adenocarcinoma cell line NCI-H322, the carcinoid cell line NCI-H727, the squamous cell lines NCI-H520 and NCI-H2170 did not express protein for α7. However, our data are in contrast to another recent study. Carlisle et al. [15] found that α7 nAChR transcripts are frequently found in human bronchial epithelial cells although the protein of the correct size is not. They also postulated that muscle-type nicotinic acetylcholine receptors might be involved in responses to nicotine. Further research is needed to determine if both neuronal and muscle type acetylcholine receptors are involved in signaling events in lung cancer cells.

Previous research from our laboratories has indicated that NNK binds with high affinity to alpha-bungarotoxin (agonist for α7 and α8 [16]) sensitive nAChRs in NCI-H69 and NCI-H82 cells, and the NNK affinity for these receptors was several times higher than for nicotine [5]. This binding was inhibited by hexamethonium but not decamethonium [5]. Hexamethonium is a selective agonist for neuronal nAChRs, whereas decamethonium is selective for muscle-type nAChRs [17]. In addition, we have found that NNK activates the Raf-1/MAP kinase pathway resulting in phosphorylation of c-myc [10]. This activation was inhibited by alpha-bungarotoxin. Lending support to this data, in the present study NNK stimulated α7 protein expression in NCI-H69 cells. Although muscle type acetylcholine receptors may be involved in signaling responses in SCLC, our data indicates the α7 neuronal nicotinic acetylcholine receptor is a functional receptor in lung cells and stimulates signaling events in these cells.

Using a nuclease protection assay that allows for direct comparisons between samples, we found higher levels of α7 nAChR mRNA receptor in NCI-H82 than in SAEC. The RT-PCR assay showing expression of α7 nAChR receptor in SAE cells is a much more sensitive assay, but does not allow for the comparisons seen in the nuclease protection assay. The increase in α7 nAChR receptor in the SCLC cell line NCI-H82 compared to the normal SAE cells from a non-smoker is consistent with other data from our laboratory and others. In addition, it has been shown that the NCI-H82 cell line synthesized the highest levels of acetylcholine, and addition of nicotinic antagonists slowed growth of the NCI-H82 cells [18]. Acetylcholine is known to act as an autocrine growth factor for SCLC [19]. This data is also consistent with protein levels of the α7 nAChR. The NCI-H82 cell line had the highest level of α7 nAChR protein of the SCLC cell lines tested.

Treatment of pregnant hamsters with the tobacco carcinogen, NNK, led to an increase in the α7 nAChR in PNEC isolated from fetal hamsters on the 15^th ^day of gestation [20]. As indicated above, previous data from our laboratory has also shown that an associated mitogenic signal transduction pathway is upregulated in SCLC. Similar results were seen in the hamster PNEC model, as protein levels of Raf-1 in NNK treated hamster PNEC were greatly increased compared to control PNEC protein levels, and the NNK treated PNEC protein levels were at similar levels to untreated SCLC cell line NCI-H69 cells [9,10]. In addition, ERK1/2 protein levels were also increased in NNK treated PNEC [9,10]. These findings are in accord with publications which have demonstrated that chronic exposure of brain cells to nicotinic agonists results in a paradoxical upregulation of this receptor [17,21-23]. Our findings are also supported by a study in monkeys, which has shown that chronic treatment of pregnant monkeys with nicotine caused a pronounced upregulation of the α7 nAChR in the lungs of the newborns [24]. Together these data indicate that the α7 nAChR may play an important role in the development of SCLC, and other lung cancers in which smoking is involved. Although there are many growth factors involved in multiple pathways leading to lung cancer, the effects of signaling through nicotinic receptors needs further investigation to determine its role in the pathogenesis of lung cancer. Further studies with lung cancer cell lines that express both the mRNA and protein for the α7 nAChR are needed.

## Conclusion

Expression of mRNA for the neuronal nicotinic acetylcholine receptor α7 seems to be ubiquitously expressed in all human lung cancer cell lines tested (except for NCI-H441) as well as normal lung cells. The α7 nicotinic receptor protein is expressed in fewer cell lines, and the tobacco carcinogen NNK increases α7 nicotinic receptor protein levels. The α7 nAChR may play an important role in the development of SCLC and other lung cancers in which smoking is involved.

## Competing interests

The author(s) declare that they have no competing interests.

## Authors' contributions

HP carried out all experiments with the exception of the western blots, participated in the design of the study, and helped draft the manuscript. MD performed the western blot experiments. HS conceived of the study and helped draft the manuscript.
